# Metabolic Variability of a Multispecies Probiotic Preparation Impacts on the Anti-inflammatory Activity

**DOI:** 10.3389/fphar.2017.00505

**Published:** 2017-07-28

**Authors:** Michele Biagioli, Luca Laghi, Adriana Carino, Sabrina Cipriani, Eleonora Distrutti, Silvia Marchianò, Carola Parolin, Paolo Scarpelli, Beatrice Vitali, Stefano Fiorucci

**Affiliations:** ^1^Department of Surgical and Biomedical Sciences, University of Perugia Perugia, Italy; ^2^Department of Agricultural and Food Sciences, Interdepartmental Centre for Agri-Food Industrial Research, University of Bologna Cesena, Italy; ^3^Department of Medicine, University of Perugia Perugia, Italy; ^4^SC di Gastroenterologia ed Epatologia, Azienda Ospedaliera di Perugia Perugia, Italy; ^5^Department of Pharmacy and Biotechnology, University of Bologna Bologna, Italy; ^6^Department of Experimental Medicine, Laboratory of Biotechnology, University of Perugia Perugia, Italy

**Keywords:** probiotics, metabolomics/metabolite profiling, inflammatory bowel diseases, animal studies, intestinal permeability, macrophage activation, cytokines and inflammation, RAGE receptor

## Abstract

**Background:** In addition to strain taxonomy, the ability of probiotics to confer beneficial effects on the host rely on a number of additional factors including epigenetic modulation of bacterial genes leading to metabolic variability and might impact on probiotic functionality.

**Aims:** To investigate metabolism and functionality of two different batches of a probiotic blend commercialized under the same name in Europe in models of intestinal inflammation.

**Methods:** Boxes of VSL#3, a probiotic mixture used in the treatment of pouchitis, were obtained from pharmacies in UK subjected to metabolomic analysis and their functionality tested in mice rendered colitis by treatment with DSS or TNBS.

**Results:** VSL#3-A (lot DM538), but not VSL#3-B (lot 507132), attenuated “clinical” signs of colitis in the DSS and TNBS models. In both models, VSL#3-A, but not VSL#3-B, reduced macroscopic scores, intestinal permeability, and expression of TNFα, IL-1β, and IL-6 mRNAs, while increased the expression of TGFβ and IL-10, occludin, and zonula occludens-1 (ZO-1) mRNAs and shifted colonic macrophages from a M1 to M2 phenotype (*P* < 0.05 vs. TNBS). In contrast, VSL#3-B failed to reduce inflammation, and worsened intestinal permeability in the DSS model (*P* < 0.001 vs. VSL#3-A). A metabolomic analysis of the two formulations allowed the identification of two specific patterns, with at least three-folds enrichment in the concentrations of four metabolites, including 1–3 dihydroxyacetone (DHA), an intermediate in the fructose metabolism, in VSL#3-B supernatants. Feeding mice with DHA, increased intestinal permeability.

**Conclusions:** Two batches of a commercially available probiotic show divergent metabolic activities. DHA, a product of probiotic metabolism, increases intestinal permeability, highlighting the complex interactions between food, microbiota, probiotics, and intestinal inflammation.

## Introduction

The World Health Organization's defined probiotics in 2001 as “live microorganisms which, when administered in adequate amounts, confer a health benefit on the host.” A wide range of microbes and applications are included in this definition and, while it captures the essence of probiotics, it is widely accepted that probiotics for which non-strain-specific claims might be made could be restricted to *Bifidobacterium* (*adolescentis, animalis, bifidum, breve*, and *longum*) and *Lactobacillus* (*acidophilus, casei, fermentum, gasseri, johnsonii, paracasei, plantarum, rhamnosus*, and *salivarius*). This list includes well-studied species which could offer some benefits if taken daily at the dose of 1 billion colony forming units (1 × 10^9^ CFU) viable cells. However, while supporting a healthy gut microbiota is the core benefit of probiotics, the current state of science does not allow for a clear definition of a healthy gut microbiota based on microbial composition (Hill et al., 2014; Bellaguarda and Chang, 2015).

Crohn's disease and ulcerative colitis are two chronic inflammatory bowel diseases (IBDs) resulting from impaired intestinal response to antigens derived from the intestinal microbiota in genetically predisposed individuals (de Souza and Fiocchi, 2016; Neurath, 2017). The IBDs have traditionally been treated with non-specific immunosuppressive drugs such as corticosteroids, anti-inflammatory agents, and more recently with inhibitors of TNFα (Mosli et al., 2014). Although these treatments have shown efficacy in mild disease, novel agents that target leukocyte trafficking to the gut by inhibiting integrins, chemokine, and chemokine receptor, have been developed but up to 40–50% of patients still fail to respond to these approaches (Mosli et al., 2014).

Probiotics have been used to treat IBDs (Chibbar and Dieleman, 2015). Among the several probiotic preparations available, VSL#3, a blend of eight different bacterial strains, has been extensively investigated and is currently recommended for the treatment of chronic pouchitis (Gionchetti et al., 2000, 2003; Shen et al., 2014). Recently, however, *in vitro* and *in vivo* studies have shown variability in the efficacy of VSL#3 and Lorén et al. have reported a lack of efficacy of VSL#3 in reducing inflammation in models of colitis (Lorén et al., 2010), while *in vitro* studies have suggested that the health-promoting properties of the VSL#3 preparations might vary form one batch to another, even when the strain taxonomy remains the same (Maassen et al., 2003; Cinque et al., 2016).

The fact that culture conditions, among other factors, impact on strain functionality is widely recognized. The IL-10-inducing activity of *L. plantarum* OLL2712 differs dramatically according to different growth phases, culture medium components, temperatures, and pH (Grangette et al., 2005; Toshimitsu et al., 2017). The exponential phase cells display enhanced IL-10-inducing activity in comparison to stationary phase cells, while the toll-like receptor (TLR)-2 stimulatory activity depends on media components and culture conditions. These biochemical effects translate into different *in vivo* properties, since cells of *L. plantarum* OLL2712 in the exponential growth phase elicit more anti-inflammatory properties than their stationary phase counterparts, firmly establishing that culture conditions impact on beneficial properties of lactic acid bacteria.

Because it has been reported (Maassen et al., 2003; Cinque et al., 2016) that at least two formulations of VSL#3 are currently available in Europe, we have challenged these preparations against two canonical models of colitis. The results of these studies revealed that only one VSL#3 mixture exerted beneficial effects, while the other had no effect or even worsened the severity of intestinal inflammation. A detailed metabolomic analysis of the two VSL#3 allowed the identification of two separate metabolomic patterns, that although largely overlapping, presented a specificity in terms of production of at least four metabolites including 1–3 dihydroxyacetone (DHA), an intermediate in the fructose metabolism. We also report that challenging mice with DHA increases intestinal permeability.

## Materials and methods

### Probiotics

Two different lots of VSL#3® were used in this study. Both lots were formulated as sachets and were commercially available in pharmacies in UK. The lot numbers and expiration dates were the following: DM538, expiration 12/2017 (indicated as VSL#3-A); and 507132, expiration 7/2017 (indicated as VSL#3-B). The manufacturer of VSL#3-A, as indicated labels, was S.I.I.T. srl (Milano, Italy), Italy; while the manufacturer of VSL#3-B is not reported. Both products were distributed in UK by Ferring Ltd, under the same brand name of VSL#3. According to the labels, each 4.4 g sachet provides a blend of 450 billion bacteria. The list of strains as they appear on their respective commercial packaging is as follow: VSL#3-A: *Streptococcus thermophilus* DSM 24731, bifidobacteria (*B. longum* DSM 24736, *B. breve* DSM 24732, *B. infantis* DSM 24737), lactobacilli (*L. acidophilus* DSM 24735, *L. plantarum* DSM 24730, *L. paracasei* DSM 24733, *L. debrueckii* subsp. *bulgaricus* DSM 24734); and *S. thermophilus* BT01, bifidobacteria (*B. breve* BB02, *B. longum* BL03, *B. infantis* BI04), lactobacilli (*L. acidophilus* BA05, *L. plantarum* BP06, *L. paracasei* BP07, *L. debrueckii* subsp. *bulgaricus* BD08) for VSL#3-B. Because the bacterial content of the two formulation is indicated by the producer we did not performed any genetic analysis on the bacteria present in the two formulations. The two batches were maintained according to the manufacturer instructions until used.

### Animals and colitis protocols

Balb/c mice were from Charles River (Italy). The colonies were maintained in the animal facility of University of Perugia. Mice were housed under controlled temperatures (22°C) and photoperiods (12:12–h light/dark cycle), allowed unrestricted access to standard mouse chow and tap water and allowed to acclimate to these conditions for at least 5 days before inclusion in an experiment. The study was conducted in agreement with the Italian law and the protocol was approved by the Ethics Committee of the University of Perugia and by a National committee of Ministry of Health (permission n. 1126/2016-PR). The health and body conditions of the animals were monitored daily by the Veterinarian in the animal facility. The study protocol caused minor suffering, however, animals losing more than 25% of the initial body weight were euthanized. Colitis was induced in Balb/c mice by administering, for 8 consecutive days, of 5% DSS (DSS: Dextran Sulfate, Sodium Salt of Affymetrix USA, molecular mass 40–50 kDa) in drinking water. Animals were monitored daily. For the TNBS colitis model, Balb/c mice were fasted for 12 h (day −1). The day after (day 0) mice were anesthetized, and a 3.5 F catheter inserted into the colon such that the tip was 4 cm proximal to the anus. To induce colitis, 1 mg of 2,4,6-trinitrobenzenesulfonic acid TNBS (Sigma Chemical Co, St Louis, MO) in 50% ethanol was administered via catheter into the lumen using a 1 ml syringe (injection volume of 100 μl); control mice received 50% ethanol alone. Animals were monitored daily. At the end of the experiments, the surviving mice were sacrificed, blood samples collected by cardiac puncture, and the colon was excised, weighed, and evaluated for macroscopic damage. In some groups of mice we administered also VSL#3-A or VSL#3-B of VSL#3 by o.s. at the concentration of 50^*^10^9^ probiotic cfu/kg of body weight dissolved in saline solution (Pagnini et al., 2010; Corridoni et al., 2012; Mencarelli et al., 2012; Distrutti et al., 2013, 2014). Both treatments were administered daily from day 0 to the day of sacrifice (Mencarelli et al., 2011, 2016). In both models the severity of colitis was measured each day for each mouse by assessing the body weight, the fecal occult blood, and stool consistency. Each parameter was scored from 0 to 4 as described in the Supplementary Table 1.

### Measurement of intestinal permeability

Eight- to ten-week old Balb-c mice, both male and female, were administered 1–3 DHA for 14 days per *os* by gavage at the following dose: 100 μL of 90 mg/ml (1 M) or 9 mg/ml (0.1 M) solution of 1–3 DHA (DHA, m.w. 90). On day 7 or 14 a permeability test was carried out using a fluorescein isothiocyanate conjugated dextran (FITC-dextran) (Sigma-Aldrich, St. Louis, MO, catalog number: FD4). We used the same reagent to study the intestinal permeability in Balb/c mice with colitis induced by DSS on day 8. FITC-dextran was dissolved in PBS at a concentration of 100 mg/ml and administered to each mouse at the dose of 44 mg/100 g body weight by oral gavage. After 4 h, mice were anesthetized by isoflurane inhalation and blood collected by cardiac puncture.

### Histology and immunohistochemistry

Samples of distal colon (2–3 cm from the anus) were first fixed in buffered formalin and routine 5 μm sections were prepared (≈150 μm between each section, 4–8 per fragment per colon) and then stained with hematoxylin and eosin *(H*&*E)*. For immunohistochemistry formalin-fixed, paraffin-embedded colon fragments were sectioned at 5 μ, deparaffinized and hydrated, then antigen retrieval was performed in water bath containing sodium citrate buffer (pH = 6) at 95–100°C for 30 min, slides were removed from the bath, allowed to cool for 20 min and then washed in PBS. After washing sections were incubated with 8% horse serum, 5 BSA and 0.2% triton for 45 min, then were incubated with primary antibodies anti ZO-1/TJP1 (diluted 1:20) made in rabbit (ThermoFisher Scientific, Massachusetts, USA) or with anti Occludin (diluted 1:35) made in rabbit (ThermoFisher Scientific, Massachusetts, USA) in PBS for 1 h. Afterwards, slides were washed three times, then incubated in secondary antibody Alexa Fluor 488 F(ab')2 fragment of goat anti rabbit IgG (H+L) (Molecular Probes, Oregon, USA) for 45 min, washed and mounted with Slow Fade Gold Antifade reagent with DAPI (Invitrogen, California, USA). Microscopy fluorescent images of tissue sections were acquired using an AxioVision.Z1 microscope equipped with an ApoTome filtering device and AxioCam mRm digital camera (Carl Zeiss Microscopy) through a 20x plan-apo objective. The ApoTome device, enabled for optical sectioning at medium setting (digital noise reduction set to off), was used to remove scattered light from out-of-focus underlying and overlying focal planes, resulting in a high axial resolution and increased signal-to-noise ratio. Images were visually optimized with a factor 140, 5 pixel sharpening mask and histograms equalization (Adobe Photoshop CC).

### Reverse transcription of mRNA and real-time (RT) PCR

rt-PCR was carried out in colon samples and mesenteric lymph nodes. The RNA was extracted using Trizol (TermoFisher Scientific) according to the manufacturer's protocol. After purification from genomic DNA by DNase-I treatment (TermoFisher Scientific), 1 μg of RNA from each sample was reverse-transcribed using random hexamer primers with Superscript-II Reverse Transcriptase (TermoFisher Scientific) in a 20 μL reaction volume; 10 ng cDNA were amplified in a 20 μl solution containing 200 nM of each primer and 10 μl of SYBR Select Master Mix (TermoFisher Scientific). All reactions were performed in triplicate, and the thermal cycling conditions were as follows: 3 min at 95°C, followed by 40 cycles of 95°C for 15 s, 56°C for 20 s and 72°C for 30 s, using a Step One Plus machine (Applied Biosystem). The relative mRNA expression was calculated accordingly to the Ct method. We studied the expression of many genes involved in inflammation: IFN-γ (for GCTTTGCAGCTCTTCCTCAT; rev ATCCTTTTGCCAGT), TNF-α (for CCACCACGCTCTTCTGTCTA; rev AGGGTCTGGGCCATAGAACT), IL-6 (for CTTCACAAGTCGGAGGCTTA; rev TTCTGCAAGTGCATCATCGT), IL-1β (for GCTGAAAGCTCTCCACCTCA; rev AGGCCACAGGTATTTTGTCG), TGF-β (for TTGCTTCAGCTCCACAGAGA; rev TGGTTGTAGAGGGCAAGGAC), IL-10 (for CCCAGAAATCAAGGAGCATT; rev CTCTTCACCTGCTCCACTGC), FoxP3 (for TCTTCGAGGAGCCAGAAGAG; rev AGCTCCCAGCTTCTCCTTTT), and Occludin (for CGGTACAGCAGCAATGGTAA; rev CTCCCCACCTGTCGTGTAGT). Furthermore, we studied the expression of markers genes for M1 and M2 macrophage populations: Cd38 (Mm01220906_m1 TermoFisher Scientific), Fpr2 (Mm00484464_s1 TermoFisher Scientific), and Egr2 (Mm00456650_m1 TermoFisher Scientific).

### ^1^H-NMR analysis

Each VSL#3 product was suspended (inoculum concentration: 1% w/v) in de Man, Rogosa and Sharpe (MRS) medium, added with L-cysteine 0.05%. The bacterial suspensions were incubated at 37°C for 44 h in anaerobic jars supplemented with GazPack EZ (Becton Dickinson and Company). At the end of the incubation period, the cultures were centrifuged at 5,000 g for 10 min and the supernatants filtered through a 0.2 μm membrane and stored at −20°C for ^1^H-NMR analysis.

Samples were prepared for NMR analysis through thawing and centrifugation for 15 min at 1500 rpm and 4°C. 0.7 ml of supernatants were added to 0.1 ml of a D_2_O solution of 3-(trimethylsilyl)-propionic-2,2,3,3-d4 acid sodium salt (TSP) 10 mM, buffered at pH 7.00 with 1 M phosphate buffer and centrifuged again.

^1^H-NMR spectra were recorded at 298 K with an AVANCE III spectrometer (Bruker, Milan, Italy) operating at a frequency of 600.13 MHz. The HOD residual signal was suppressed by means of presaturation. Following the procedure of Parolin et al. (2015), the signals from broad resonances originating from large molecules were suppressed by a CPMG-filter, while the HOD residual signal was suppressed by means of presaturation. Each spectrum was acquired by summing up 256 transients using 32 K data points over a 7,184 Hz (for an acquisition time of 2.28 s). NMR analysis was carried out according to a previously described method (Barbara et al., 2016). Briefly, the recycle delay was set to 5 s, keeping into consideration the longitudinal relaxation time of the protons under investigation. The signals were assigned by comparing their chemical shift and multiplicity with Chenomx software data bank (Chenomx Inc., Canada, ver. 8.1).

### Data analysis

^1^H-NMR spectra were baseline-adjusted by means of the simultaneous peak detection and baseline correction algorithm (SPDBC) implemented in the R package baseline (Liland et al., 2010). Quantification of the molecules was performed in pure MRS sample by employing the added TSP as internal standard. In order to compensate for differences in dilution or solids content, any other sample was then normalized to pure MRS sample by means of probabilistic quotient normalization (Dieterle et al., 2006). The concentration of all the profiled molecules was expressed as mmol/L. For animal studies a one way ANOVA followed by Bonferroni and *a t*wo-tailed unpaired Student *t* test was used for statistical comparisons (^*^P < 0.05) using the Prism 6.0 software (GraphPad).

## Results

### VSL#3 batches

The two lots used in this study were commercially available in UK and were code-named VSL#3-A and VSL#3-B. Each batch was maintained at 4°C and handled according to the manufacturer instructions.

### DSS colitis

**Figure 1:**
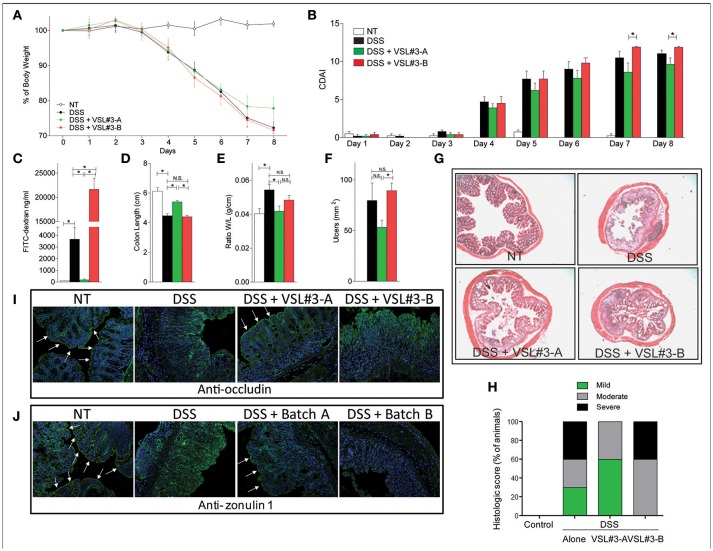
Effects of VSL#3 on DSS colitis. Mice were treated with DSS in drinking water and then administered with vehicle or one of the two formulations of VSL#3 by gavage from day 0 to day 8. VSL#3-A attenuated the development of wasting disease, i.e., change in body weight **(A)** and CDAI score **(B)**. VSL#3-B worsens of intestinal permeability: FITC dextran **(C)**. **(D–F)** Only VSL#3-A reduced intestinal inflammatory score: colon length **(D)**, ratio between colon weight and colon length **(E)**, and ulcers area **(F)**. H&E staining on colon sections **(G)** and analysis of histological score **(H)** of control, DSS treated and DSS plus one of the two formulations of VSL#3 (magnification 10x). Representative immunohistochemistry of colon with anti-occludin **(I)** and anti-zonulin1 **(J)** antibody for each experimental group. Results are the mean ± SEM of 4–6 mice per group (^*^P < 0.05).

Because VSL#3 modulates the integrity of intestinal mucosal barrier, we have first investigated whether the two batches effectively reduce intestinal damage caused by feeding mice with DSS in drinking water. As illustrated in Figure 1, both VSL#3-A and B were poorly effective in protecting against colitis development in this model. However, a significant recovery in body weight (Figure 1A) and CDAI score (Figure 1B) was observed with VSL#3-A starting from day 7 of administration, while treating DSS-mice with VSL#3-B failed to improve signs and symptoms of colitis.

Measurement of intestinal permeability using a FITC-dextran (Figure 1C) demonstrated that, while VSL#3-A reversed the damaging effect of DSS on the intestinal barrier, VSL#3-B did the opposite and increased the intestinal permeability ≈10-folds in comparison to DSS alone (*P* < 0.01 vs. DSS alone and DSS plus VSL#3-A). These somewhat unexpected results were confirmed at the necroscopy investigation of colons performed at the end of the study. Again, while VSL#3-A attenuated the effect of inflammation on colon length (Figure 1D), on the ratio between weight and length of the colon (Figure 1E) and number and extent of colonic ulcerations (Figure 1F), these beneficial effects were lost in mice treated with VSL#3-B (Figures 1D–F). The histologic examination of colon sections stained with H&E demonstrated that treating DSS mice with VSL#3-A attenuated the severity of inflammation and leukocyte infiltration, while no improvement observed in mice administered VSL#3-B which increased the severity of colitis (Figures 1G,H). Moreover, the immunohistochemistry analysis of occludin (Figure 1I) and zonula occludens-1 (ZO-1) (Figure 1J), revealed that while DSS caused a disruption of intestinal tight junctions and severely reduced the expression and tissue distribution of both proteins, this pattern was reversed by treating mice with VSL#3-A. In contrast, no beneficial effects were observed in mice administered VSL#3-B, highlighting a striking correlation between changes in intestinal permeability and expression of tight-junction proteins.

### TNBS colitis

**Figure 2:**
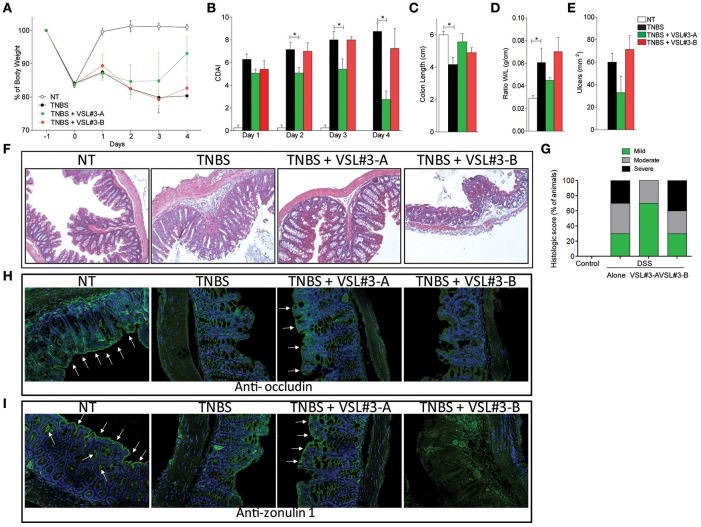
Effects of VSL#3 on TNBS colitis. Mice were treated with TNBS and then administered with vehicle or one of the two formulations of VSL#3 by gavage from day 0 to day 4. VSL#3-A attenuated the development of wasting disease, i.e., change in body weight **(A)** and CDAI score **(B)**. **(C–E)** Only VSL#3-A reduced intestinal inflammatory score: colon length **(C)**, ratio between colon weight and colon length **(D)**, and ulcers area **(E)**. H&E staining on colon sections **(F)** and analysis of histological score **(G)** of control, TNBS treated, and TNBS plus one of the two formulations of VSL#3 (10x). Representative immunohistochemistry of colon with anti-occludin **(H)** and anti-zonulin1 **(I)** antibody for each experimental group. Results are the mean ± SEM of 4–7 mice per group (^*^P < 0.05).

**Figure 3:**
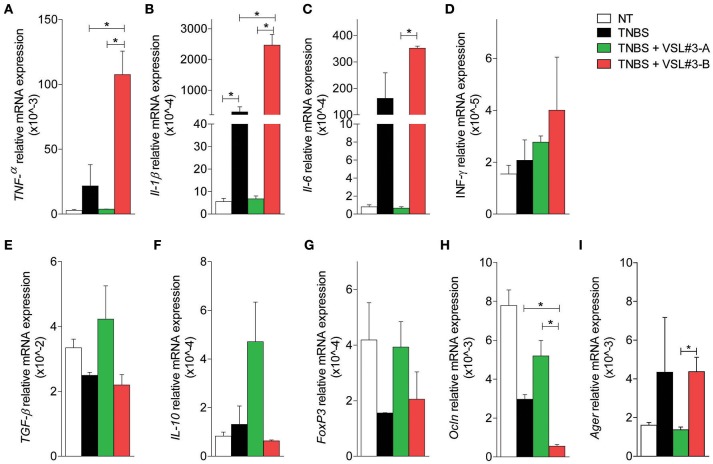
Effects of two VSL#3 preparations on biomarkers of intestinal inflammation in the TNBS colitis. Quantitative rtPCR analysis of pro-inflammatory genes TNF-α, IL-1β, IL-6 and IFN-γ **(A–D)**, anti-inflammatory genes, TGF-β, IL-10, and FoxP3**(E–G)**, occludin **(H)**, and Ager **(I)**. Results are the mean ± SEM of 4–7 mice per group (^*^P < 0.05).

To gain further mechanistic insights on the effects exerted by the two VSL#3 batches, and taking into consideration that DSS exerts a profound inhibitory effect on enzyme used in PCR analysis, we carried out a second set of experiments in mice administered with TNBS, a widely used model of Th1-mediated disease. Again, while the severity of colitis was significantly reduced by treating mice with VSL#3-A (Figures 2A,B), VSL#3-B failed to achieve the same results. These findings were confirmed at pathology analysis, since VSL#3-A protected from the effects of TNBS on colon length, weight and ulcerations, while VSL#3-B had no effect (Figures 2C–E). Further, VSL#3-A reduced the extent of inflammatory infiltrate and epithelial destruction (Figure 2F) and the severity of histology scores (Figure 2G), but these protective effects were lost in TNBS mice administered VSL#3-B (Figures 2F,G). These findings were confirmed by immunohistochemistry analysis of occludin and ZO-1 (Figures 2H,I, respectively). Indeed, while exposure to TNBS decreased the expression of both proteins and this pattern was reversed by VSL#3-A, the expression of occludin and ZO-1 was almost undetectable in the colon of the animals treated with VSL#3-B. Confirming the above mentioned findings, while VSL#3-A reduced the expression of TNFα, IL1β, IL6 mRNAs (Figures 3A–D) and increased the expression of anti-inflammatory genes, i.e., TGFβ, IL-10, and FoxP3 (Figures 3E–G), the opposite was observed in TNBS-mice treated with VSL#3-B, since the latter boosted the expression of pro-inflammatory cytokines, causing a three-fold increase in TNFα and IL-6 mRNAs in comparison with TNBS alone and a 50-fold increase of IL-1β mRNA (Figure 3A; *P* < 0.01 vs. VSL#3-A). Furthermore, VSL#3-B had no effects on the expression of TGFβ and IL10, thought that similarly to VSL#3-A, it slightly increased the expression of FoxP3 (Figure 3G). Moreover, while exposure to TNBS reduced the expression of occludin this pattern was fully reverted by treatment with the VSL#3-A, while VSL#3-B further impaired the expression of this gene (Figure 3H). In addition, while TNBS administration increased the expression of Ager in the colon, this pattern was completely reversed by VSL#3-A treatment. In contrast, the administration of VSL#3-B did not affect the expression of the Ager gene (Figure 3I).

**Figure 4:**
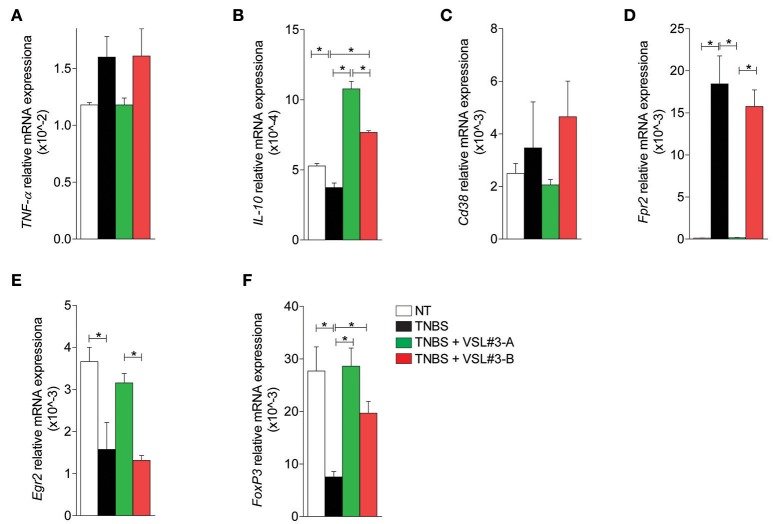
Effects of two VSL#3 preparations on biomarkers of intestinal inflammation in the TNBS colitis. Quantitative rtPCR analysis on mesenteric lymph nodes: pro-inflammatory cytokine TNF-α **(A)**, anti-inflammatory cytokine IL10 **(B)**, M1 macrophage markers Cd38 and Fpr2, **(C,D)**, M2 macrophage marker Egr2 **(E)**, and Treg cells marker FoxP3 **(F)**. Results are the mean ± SEM of 3–5 mice per group (^*^P < 0.05).

Finally, investigation of the expression of cytokines and markers of macrophages and Treg cells in the mesenteric lymph nodes, confirmed these findings. While upon colitis induction, TNBS increased the expression of TNF-α and markers of a M1 phenotype and reduced the expression of IL-10, and markers of M2 phenotype and FoxP3 (Figures 4A–F), this pattern was fully reversed by treatment with VSL#3-A, while VSL#3-B had no effect.

In an additional experiment we have compared the anti-inflammatory activity of the two VSL#3 preparations to a steroid: the results demonstrate that in the TNBS model VSL#3-A was approximately 30% less effective than dexamethasone (5 mg/kg) in reducing the “clinical” signs of colitis, while VSL#3-B had no effect (Supplementary Figure 1).

### Metabolomic analysis

To gain insights on reasons that might explain the above described variability in probiotic functionality *in vivo*, we have investigated the metabolism of the two bacterial mixtures. Studies carried out in cell supernatants of VSL#3-A and -B, cultured for 44 h using a ^1^H-NMR allowed the identification of 23 metabolites mainly pertaining to the classes of amino acids, short chain fatty acids, organic acids, monomeric carbohydrates, and nucleobases. While the two metabolomics patterns were highly overlapping, there were at least four metabolites that showed a variation >30% between VSL#3-A and -B, i.e., pyruvate, uracil, 1–3 DHA, and uridine with the last two showing a three-folds enrichment in samples obtained from VSL#3-B in comparison with VSL#3-A (Table 1).

**Table 1:** Concentration of the molecules identified in MRS and VSL#3 cultures incubated for 44 h (mmol/l of suspension), which showed a difference of at least 30% between the two samples under consideration after incubation.

	**Pure MRS**	**Batch A**	**Batch B**	A/B^*^100
Acetoin	1.77 × 10^−2^	3.53 × 10^−1^	2.69 × 10^−1^	131
sn-glycero-3-phosphocholine	5.15 × 10^−2^	1.28 × 10^−1^	9.50 × 10^−2^	135
1,3-Dihydroxyacetone	6.42 × 10^−2^	2.79 × 10^−2^	2.15 × 10^−1^	13
Glucose	19.9	1.37	2.14	64
Uracil	9.57 × 10^−2^	9.77 × 10^−2^	6.37 × 10^−2^	153
Formate	6.02 × 10^−2^	4.59 × 10^−2^	3.06 × 10^−2^	150

### Effect of DHA on intestinal permeability

**Figure 5:**
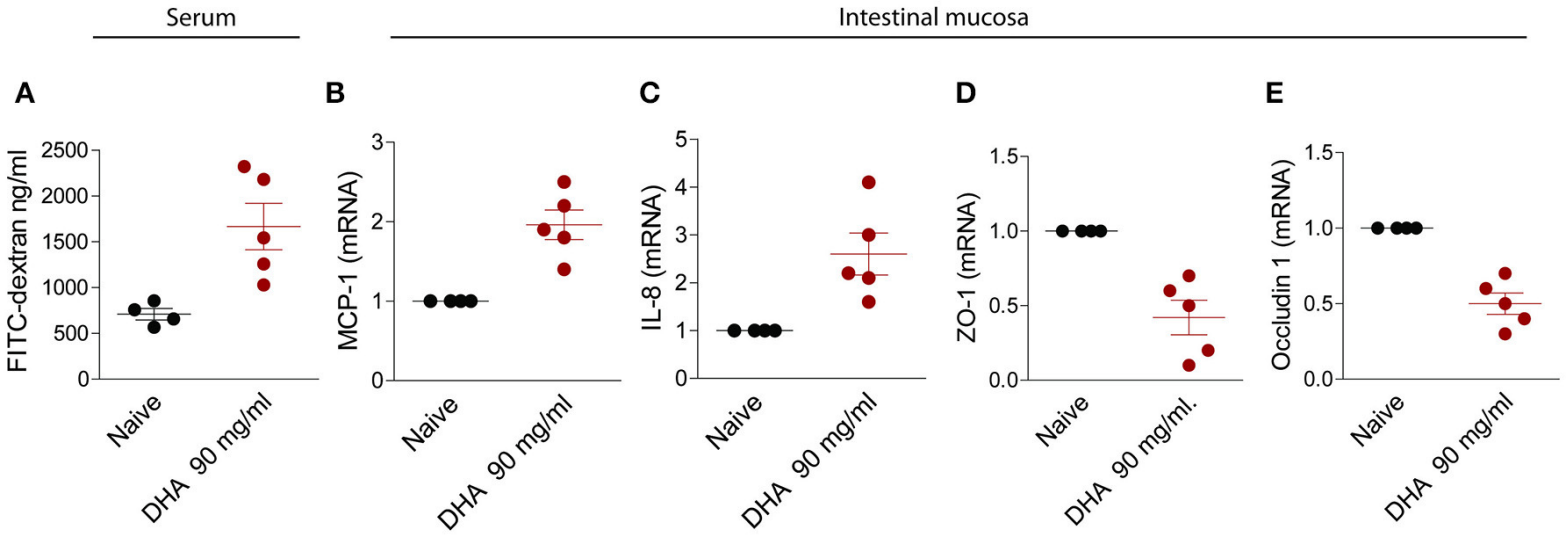
Effect of feeding mice with a high dose DHA, 90 mg/ml/day. Intestinal permeability was measured after 2 weeks of diet with FITC-dextran **(A)**. Quantitative rtPCR analysis of MCP-1 **(B)**, IL-8 **(C)**, zonulin1 **(D)**, and occludin1 **(E)**. Data are the mean ± SEM of five mice per group (^*^P < 0.05).

Because DHA is part of fructose metabolism, we have then investigated whether feeding mice with this agent reproduced the same effects observed in mice administered VSL#3-B on intestinal permeability. Results shown in Figure 5, demonstrated that feeding DHA in drinking water, dose-dependently increased the intestinal permeability. While no effects were observed in mice feed 9 mg/kg day of DHA, a 10 fold higher dose increased significantly both the intestinal permeability and the markers of inflammation and reduced the expression of ZO-1 and occludin mRNA.

## Discussion

The presented study was prompted to us by recent reports which have described a variability in the functionality of VSL#3, a widely used probiotic. Cinque et al. have recently shown that batches of VSL#3 obtained from different European countries differ in number of living vs. dead cells ratio, cell division properties and some *in vitro* functionality (Cinque et al., 2016). Additionally, it has been reported that VSL#3 had no effect on colitis induced by DSS. In the present study we report that two batches of VSL#3 distributed in UK vary considerably in terms of their functionality in two models of colitis. Since we have not performed any genetic study, we are unable to establish whether this variability is due to a different strain taxonomy.

It is known that the health-promoting properties of probiotics change widely in response to their culture conditions, even when the strain remains the same (Toshimitsu et al., 2017). Changes in culture conditions impact bacteria metabolism which may results in profound variability in probiotic functionality. Toshimitsu et al. have shown that the IL-10 and IL-12 modulatory activity of *L. plantarum* OLL2712 changes according to culture conditions and media composition. Similar findings were reported by Sashihara et al. (2007) that have shown that stationary phase cells of *L. gasseri* strain stimulates murine splenocytes to secrete higher amount of IL-12 than exponential phase cells. Similarly *L. plantarum* WCFS1 cells in the two phases, differently modulated peripheral blood mononuclear cells to secrete IL-10 and IL-12 (van Baarlen et al., 2008). Taken together these data indicate that culture conditions widely affect anti-inflammatory activity of probiotic.

Even the composition media, seems to have a major impact. Indeed, previous studies have shown that oleic acid affected the cell growth (Endo et al., 2006) or survival in the gastric juice (Corcoran et al., 2007) of some *lactobacilli* strains by being incorporated into bacterial cell surface membrane lipids. Toshimitsu et al. (2017) have reported that decaglycerol mono-oleic acid 1 ester Q-17S, normally used as an emulsifier for food, affected the immunomodulatory properties of *L. plantarum* strains, while the same effects were not observed with decaglycerol mono-stearic acid ester Q-18S, highlighting the fact that subtle changes in culture media deeply impact on structure of bacterial cell components and functionality.

Because culture details of the two VSL#3 strains were not provided, we are unable to establish whether different culture settings might explain the different functionality we have observed in this study.

Nevertheless, the metabolomic analysis of VSL#3-A and -B cultures gives rise to two specific patterns. Despite the fact that culture conditions and media were the same, the two probiotic blends generated a distinct pattern of metabolites. Over 23 metabolites that were examined, 19 showed no variations, but concentrations of four metabolites, pyruvate, DHA, uracil, and uridine were significantly higher (>three-folds) in supernatants of VSL#3-B. While all these metabolites deserve attention, we have focused our attention on DHA. DHA and DHA phosphate (DHAP) are inter-convertible metabolites generated when fructose 1,6-bisphosphate breaks down into DHAP and glyceraldehyde 3-phosphate. In some circumstances, DHA can be further metabolized to form glyoxal and methylglyoxal (MG). Once formed, MG principally reacts with arginine groups to form advanced glycation end products (AGEs). MG levels can be regulated via the activity of two enzymes, glyoxalase I and II (Thornalley, 2003), that detoxify MG and convert it into d-lactate (Phillips and Thornalley, 1993). Exogenous sources of AGEs contribute to AGE formation *in vivo*. Foods high in fat contain AGEs, along with the AGE precursor, MG. An additional deposit for the formation of these products is represented by dietary and environmental sources of AGEs; hence AGE levels and their consequences may be amplified when detoxification mechanisms, such as glyoxalases, or clearance mechanisms are impaired (Bierhaus and Nawroth, 2009).

In the present study we have shown that intestinal inflammation induced by TNBS increases the expression of Ager (SCARJ1), a gene that encodes for the AGE receptor (RAGE). Ager is a member of the immunoglobulin superfamily of cell surface receptors (Fritz, 2011). This gene encodes for nine different isoforms of RAGE in humans and four in the mouse (Fritz, 2011). The interaction between RAGE with its ligands results in the activation of pro-inflammatory genes with generation of key proinflammatory mediators including NF-κB (Ohgami et al., 2001; Fritz, 2011). RAGE, has a role in intestinal inflammation in both Crohn's disease and ulcerative colitis, (Andrassy et al., 2006) and protein extracts from gut specimens from patients with IBDs cause NF-κB activation in cultured endothelial cells and causes intestinal inflammation and NF-κB activation when administered intrarectally to wild-type mice, but fails to do so in RAGE^−/−^ mice (Andrassy et al., 2006).

Of relevance, we have observed that while VSL#3-A reduced the intestinal expression of Ager, VSL#3-B did not, either suggesting lack of anti-inflammatory activity of the latter probiotic blend or production of RAGE ligands *in vivo*. Despite the fact that we have not investigated whether DHA converts *in vivo* into glycoxal or MG, feeding mice with a high dose of DHA for 2 weeks increased intestinal permeability (Obeid et al., 2005). Whether these metabolomic studies provide a clue for the effects we have observed *in vivo* is unclear, and further investigations are needed (Obeid et al., 2006).

In conclusion, we have shown that two batches of the same probiotic blend available in UK have different functionality *in vivo* and *in vitro*. Additionally, we have shown that DHA, a metabolite generated by bacterial metabolism, increases intestinal permeability, highlighting the complex interactions between food, intestinal microbiota, probiotics, and inflammation.

## Author contributions

MB, SC, AC, and SM: carried out animal studies, molecular biology analysis and histopathology, and co-wrote the manuscript; LL, CP, BV: carried out metabolomic studies, elaborate statistic of metabolomic data, and co-wrote the manuscript; PS: carried out immunohistochemistry studies and co-wrote the manuscript; ED and SF: conceived the study and wrote the manuscript.

### Conflict of interest statement

The authors declare that the research was conducted in the absence of any commercial or financial relationships that could be construed as a potential conflict of interest.

## Supplementary material

Supplementary Figure 1 Balb/c mice were treated with TNBS and then administered with VSL#3-A, VSL#3-B, or dexamethasone 5 mg/kg/day. Changes in body weight **(A)**. CDAI score **(B)**. Results are the mean ± SEM of 8–14 mice per group from two independent experiments (^*^*P* < 0.05).

Supplementary Table 1 Calculation of CDAI (Colitis Disease Activity Index) score.
