# Supplementary figures and images for: Metabolic Variability of a Multispecies Probiotic Preparation Impacts on the Anti-inflammatory Activity
Source: Front Pharmacol. 2017 Jul 28;8:505. doi: 10.3389/fphar.2017.00505 (PMC5532379; doi:10.3389/fphar.2017.00505)

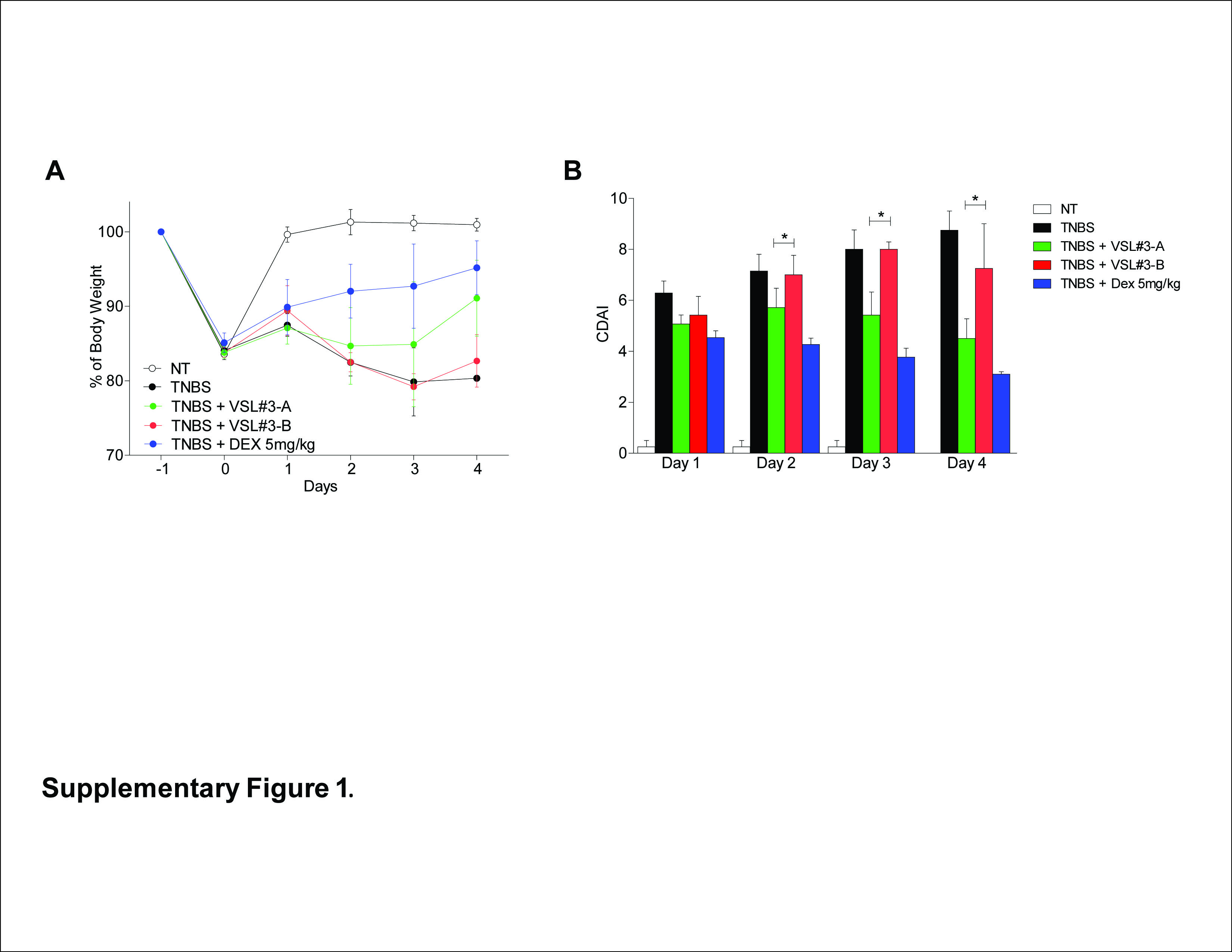

Supplement: Supplementary Figure 1 — Balb/c mice were treated with TNBS and then administered with VSL#3-A, VSL#3-B, or dexamethasone 5 mg/kg/day. Changes in body weight (A). CDAI score (B). Results are the mean ± SEM of 8–14 mice per group from two independent experiments (*P < 0.05). [file Image1.JPEG]
