# Supplementary material for: Metabolic Variability of a Multispecies Probiotic Preparation Impacts on the Anti-inflammatory Activity
Source: Front Pharmacol. 2017 Jul 28;8:505. doi: 10.3389/fphar.2017.00505 (PMC5532379; doi:10.3389/fphar.2017.00505)
Supplement: Supplementary Table 1 — Calculation of CDAI (Colitis Disease Activity Index) score. [file Table1.DOC]

**Supplementary Table 1**

| **Score** | **% of weight loss** | **Stool consistency** | **Occult bleeding** |
| --- | --- | --- | --- |
| 0 | None | Normal | None |
| 1 | 1-5 | Soft but still formed |  |
| 2 | 5-10 | Very soft | Visible in the stool |
| 3 | 10-20 | Diarrhea |  |
| 4 | >20 | Liquid stools that stick to the anus or anal occlusion | Severe bleeding with fresh blood around the anus and very present in the stool blood. |
